# Serum cardiovascular-related metabolites disturbance exposed to different heavy metal exposure scenarios

**DOI:** 10.1016/j.jhazmat.2021.125590

**Published:** 2021-08-05

**Authors:** Feifei Liu, Xiaolu Chen, Yisi Liu, Zhiping Niu, Hong Tang, Shuyuan Mao, Na Li, Gongbo Chen, Hao Xiang

**Affiliations:** a Department of Global Health, School of Health Sciences, Wuhan University, 115# Donghu Road, Wuhan 430071, China; b Global Health Institute, School of Health Sciences, Wuhan University, 115# Donghu Road, Wuhan 430071, China; c Department of Environmental and Occupational Health Sciences, University of Washington, Seattle, WA 98105, USA; d Department of Occupational and Environmental Health, School of Public Health, Sun Yat-sen University, Guangzhou 510080, China; e Guangdong Provincial Engineering Technology Research Center of Environmental and Health risk Assessment, School of Public Health, Sun Yat-sen University, Guangzhou 510080, China

**Keywords:** Particulate matter, Components, Acute effects, Metabolism, Health risk assessment

## Abstract

Health effects induced by heavy metal components of particulate matter need further research. A total of 32 healthy volunteers were recruited to walk for 4 h in two different exposure scenarios in Wuhan from May 1 to Jun 30, 2019. Metabolomics technology was used to identify serum cardiovascular-related metabolites disturbance, and the health risk assessment model was employed to assess the non-carcinogenic and carcinogenic risks associated with airborne heavy metals. The results showed that the average mass concentrations of Co, Ni, Cd, Cu, Ag and Ba in PM_10_ from May 1 to Jun 30, 2019 were 0.22, 0.49, 11.53, 2.23, 34.47 and 4.19 ng/m^3^, respectively, and were 0.86, 128.47, 291.85, 291.94, 98.55 and 422.62 ng/m^3^ in PM_2.5_, respectively. Healthy young adults briefly exposed to heavy metals were associated with serum cardiovascular-related metabolites disturbance, including increased SM(d18:1/17:0) and Sphingomyelin, and decreased GlcCer(d16:1/18:0) and Galabiosylceramide, simultaneously accompanied by activation of the sphingolipid metabolism pathway. Non-carcinogenic and carcinogenic risks of airborne heavy metals via the inhalation route were observed, Ni and Cd most influenced to potential health risks. Findings indicated exposure to increment of heavy metals may increase health risks by causing cardiovascular-related metabolites disturbance via activating the sphingolipid metabolism pathway.

## Highlights

• Heavy metal exposures were associated with serum metabolites disturbance.
• Sphingolipid metabolism pathway plays key role in mediating heavy metals’ effects.
• Non-carcinogenic risks of Ni and Cd in particulate matters were observed.
• Cd in PM_2.5_ has potential risks of developing cancers among adults.

## Graphical Abstract

**Figure:**
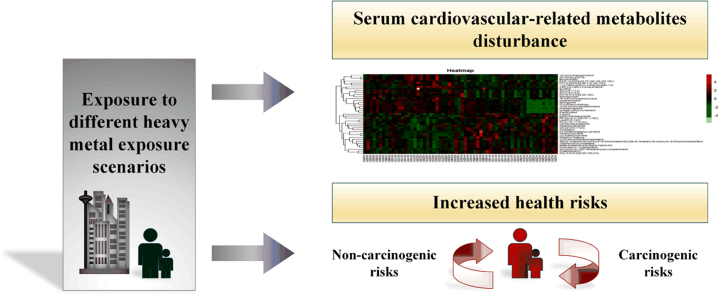

## 1 Introduction

Heavy metal exposures pose a significant threat to human health (Wang et al., 2020), which has been identified as risk factor for cancer and non-cancerous disease (e.g., cardiovascular, metabolic disease) (Choi et al., 2020, Ledda et al., 2020, Domingo-Relloso et al., 2019). Particularly, some heavy metals (e.g., Ni, Cd) have been classified as Class I carcinogen to humans (IARC (International Agency for Research on Cancer), 2011). The main source of heavy metals is metal smelting. Emitted heavy metals from different smelters are transferred to air pollutants (Gao et al., 2019), which can easily enter the body via inhalation route (Cai et al., 2019). Previous researches have provided convincible evidences that heavy metals are common in air pollutants (Marx et al., 2014, Wang et al., 2020), and are main components of particulate matter (PM) (Li et al.). However, health effects of heavy metals in PM and the underlying mechanisms have not been well studied.

Metabolomics technology can be used to identify early health effects of heavy metals in PM and to reveal the underlying mechanisms, which has been applied in metabolic disturbance research associated with PM exposures (Du et al., 2020, Zhang et al., 2019). For example, a quasi-experiment during the Beijing Olympics showed significant differences in serum metabolites between high-exposure and low-exposure periods (Mu et al., 2019). A crossover trial in Shanghai reported exposure to PM_2.5_ was correlated with obvious changes in both serum and urine metabolites (Li et al., 2017a, Chen et al., 2019). However, it still remained unclear how PM caused metabolic disturbance. Some studies indicated heavy metal components of PM may induce systemic inflammation and oxidative stress, leading to metabolites changes (Chung et al., 2020, Ku et al., 2017). To date, few studies used metabolomics to explore potential mechanisms of metabolites disturbance exposed to PM-bound heavy metals.

Health risk assessment can provide evidence for policy-making regarding the environment and human health protection, comprehensive evaluations of human health risks connected with PM-bound heavy metals are also warranted. Inhalation is the major exposure route of heavy metals in PM. However, results of existing studies on human health risks of airborne heavy metals via the inhalation route were inconsistent. In particular, studies in China showed great regional differences. For example, carcinogenic risks of Ni and Cr in PM_2.5_ through the inhalation route for adults in Beijing were 4.6E-05 and 5.30E-03, respectively (Cui et al., 2020), while those were 1.73E-01 and 1.09E-01 in Nanjing (Hu et al., 2012). Regional differences were also reported elsewhere (Gao et al., 2015). However, the comprehensive health risks assessment of heavy metal components in PM is not available in Wuhan, a representative city in central China.

Wuhan is a traffic-hub in central China, operating well-developed railway, highway and waterway transportation. Ambient PM in Wuhan is mainly from urban (e.g. traffic emissions, construction or demolition) and industrial emissions (Querol et al., 2006, Acciai et al., 2017). According to the annual report of Wuhan vehicle emission control, PM_10_ and PM_2.5_ emitted by vehicles in Wuhan were 1300 and 12,200 tons in 2018 (Bureau, 2018).

To better understand early health effects of heavy metals in PM and the underlying mechanisms, we conducted a panel study in Wuhan from May 1 to Jun 30, 2019, where 32 healthy young adults walked for 4 hours in different exposure scenarios. Health risks of heavy metal components in PM_10_ and PM_2.5_ were evaluated simultaneously based on the model of health risk assessment.

## 2 Materials and methods

### 2.1 Population collection

Healthy college students age 20–29 years were recruited via an online advertisement from the School of Medicine, Wuhan University. All participants were non-smokers and had studied at Wuhan University for at least two years. Ineligible participants were those who: (1) went to other cities within the past three months; (2) lived out of the campus within the past two weeks; (3) had physician-diagnosed hypertension, diabetes, pre-diabetic states and/or other metabolic diseases; (4) had history of allergic disease; (5) had erratic eating habits, diet or food hypersensitivity; (6) took hormones and/or anti-inflammatory medications and/or took any dietary supplement within the past six months; (7) drunk at least 12 times per year within the past three years; (8) BMI ≥ 30.0 kg/m^2^. Forty-seven volunteers were screened in our study, according to inclusive and exclusive criteria listed above, finally, 32 eligible participants were included in this study.

### 2.2 Study design

We conducted a panel study in two different regions, The Moon Lake Park and Zhongyuan Square of Wuhan, from May 1 to June 30, 2019. The Moon Lake Park has large green space and represented a low-exposure scenario, while the Zhongyuan Square was close to heavy traffic and represented a high-exposure scenario. Participants were first arranged to the Moon Lake Park from the School of Medicine by new-energy vehicles. Each participant was asked to walk at a steady pace for 4 hours (8:00–12:00) along the Moon Lake Park, and to take a break every 30 mins. After the 4 hours walk, participants returned to the School of Medicine by new-energy vehicles within 15 mins. The car windows and air conditioner were kept closed during the ride of going and returning. When arrived, blood samples (10 mL) were collected from participants by the physician immediately, and were processed for biomarkers measurements. Rest blood samples were transferred to −80 °C within 20 mins. After a 2-week washout period, participants were arranged to the Zhongyuan square. The research process was consistent with the low-exposure entirely. This work has received approval for research ethics from the Wuhan university and a proof of approval is available upon request. Each participant provided informed consent.

### 2.3 Heavy metal measurements

Mass concentrations of six heavy metal components in the Moon Lake Park and Zhongyuan Square were measured hourly by two fixed real-time multi-metals monitors of Wuhan atmospheric supersite. The monitor at the Moon Lake Park, surrounding by lawns and shrubs, located at 30.55°N and 114.26°E. The sources of heavy metal components were less affected by the human activities. The latitude and longitude of another monitor at Zhongyuan square were 30.62°N and 114.38°E, respectively. This site was surrounded by commercial properties and residential dwellings, and was close to heavy traffic. The sources of heavy metal components were mainly human ordinary activities and vehicle emissions. Heavy metal components in PM were collected and measured using the ambient heavy metals online monitoring instruments which include ambient PM enrichment system (fitted with PM_10_ or PM_2.5_ separate device), roll film system, X-ray fluorescence (XRF) analysis system, electronic display and control system etc (TH-2016, TianHong, Wuhan, China). In brief, PM sampling was separate by PM_10_ or PM_2.5_ separate device, subsequently PM_10_ or PM_2.5_ was sampled onto an automated polytetrafluoroethylene (PTEF) filter tape of the roll film system with a time interval of one hour between each subsequent sample. The sampled filter tape then moved forward to the XRF analysis system where heavy metal components in PM_10_ or PM_2.5_ were measured at high temporal resolution. The obtained spectra of heavy metals were then analysed and calibrated using the electronic display and control system, with the mass concentrations of heavy metals exported online finally. In addition, concentrations of heavy metals below the limit of detection (LODs) were recorded as 0. In addition, hourly PM_10_, PM_2.5_, temperature and relative humidity of each exposure site were also collected.

### 2.4 Biomarkers measurements

Cardiovascular biomarkers were measured in our study, including biomarkers for systemic and cardiovascular inflammation (Blood parameters: white blood cell count (WBC) and red blood cell count (RBC), concentration of hemoglobin (HGB), count of platelet (PLT), absolute neutrophil (ANC), absolute lymphocyte (LYM) and absolute monocyte (MONO); Hyper-sensitive C-reactive protein (hs-CRP) concentration), the systolic pressure (SBP), diastolic pressure (DBP), as well as heart rate (HR) (Acciai et al., 2017, Bureau, 2018). Blood parameters were measured using the Bayer ADVIA-120 automatic blood analyzer (Germany). Levels of hs-CRP were measured using the Roche/Hitachi MODULAR automated analyzer (Switzerland). SBP, DBP and HR were measured at the end of the walk by trained workers using the OMRON electronic sphygmomanometer (China).

### 2.5 Serum metabolites measurement

Untargeted metabolomics of serum samples was performed to identify changes of serum metabolites, using an ultra performance liquid chromatography-tandem mass spectrometry (UPLC-MS) metabolomics platform. We summarized the work process of UPLC-MS as following: (1) Serum samples preparation: serum samples removed from −80 °C refrigerator and were thawed on ice. Each 100 μL serum sample was treated with 20 μL internal standards and 300 μL mixture of methanol and acetonitrile. Subsequently vortexed the mixture and centrifuged (13,000 rpm, 10 mins) to remove precipitated proteins. (2) Metabolic feature detection: sample supernatants were measured in triplicate using UPLC-MS technique (Dionex Ultimate 3000 and Q-Exactive mass spectrometer) in both positive and negative ionization modes to detect the serum metabolic feature. (3) Data preprocessing: including data filtering, peaks identification, deconvolution for detected peaks, retention time (RT), peak intensity alignment and metabolic feature annotation. Ultimately, only peaks with missing value in less than 50% of serum samples were retained for further analysis. Human metabolome database and Lipid maps databases were used in the annotation (Want, 2018, Schrimpe-Rutledge et al., 2016). Moreover, data quality control was performed as well. A pooled serum sample was used as the quality control samples for reproducibility eﬀect evaluation. Relative standard deviation less than 0.4 among quality control samples with the score of qualitative analysis less than 30 points were excluded. After data cleaning, the zero-values of metabolic features were replaced with one-half of the lowest value detected.

### 2.6 Statistical analysis

Levels of cardiovascular biomarkers were log-transformed and compared using a paired-*t*-test to compare the change of each biomarker between the high- and low-exposure scenario. Biomarkers with p values of paired *t*-test less than 0.05 were selected as significant cardiovascular biomarkers for further analysis.

We conducted principal component analysis (PCA) to examine clustering of log-transformed metabolic features. Besides, the orthogonal partial least squares-discriminant analysis (OPLS-DA) was performed to evaluate separation of serum metabolic features between the 2 different exposure scenarios. For each metabolite feature with the value of variable influence on projection (VIP) in the OPLS-DA model larger than 1.0, we ﬁtted a linear mixed effect model, with log-transformed levels of each significant cardiovascular biomarker as a continuous outcome variable, to identify metabolites related to significant cardiovascular biomarkers (Model 1). Age, gender and BMI were adjusted as ﬁxed-eﬀect terms, individual ID code and exposure (1 for low-exposure scenario and 2 for high-exposure scenario) were adjusted as random effect terms. For metabolites related to 1 or more significant cardiovascular biomarkers, we ﬁtted another linear mixed effect model to evaluate metabolite differences between the 2 different exposure scenarios, with each metabolite as the outcome variable and exposure scenario as a binary independent variable (Model 2). In the model 2, age, gender and BMI were adjusted as ﬁxed-eﬀect terms, individual ID code was adjusted as random effect term. The false discovery rates (FDR) of results of two multiple comparisons (model 1 and model 2) were calculated using the Benjamini-Hochberg method.

Metabolite features were considered as differential metabolites when (1) VIP was larger than 1.0; (2) related to 1 or more significant cardiovascular biomarkers (p value in the model 1 less than 0.05 and FDR less than 0.05); (3) changed significantly between the two different exposure scenarios (p value in the model 2 less than 0.05 and FDR less than 0.05). These identified differential metabolites were considered to be associated with cardiovascular-related early health effects of heavy metal exposures. The fold change (FC) of the average expression of differential metabolites between the high- and low-exposure scenario was calculated. We also presented the expression abundance of differential metabolites using a heatmap.

Pathway analysis was performed using the KEGG database (https://www.kegg.jp/) based on differential metabolites. Hypergeometric test was performed to identify the significantly enriched pathways, and FDR of results of hypergeometric test was calculated using the Benjamini-Hochberg method. Pathway with p value from hypergeometric test less than 0.05 and FDR less than 0.05 was interpreted in the study.

The strengthening the reporting of observational studies in epidemiology (STROBE) Checklist (including 22 items that relate to the study design, conduct, and analysis) was used as a guideline to ensure quality of the study (See Table A.1).

### 2.7 Health risks assessment

All residents living in Wuhan are potentially exposed to heavy metal components in PM_10_ and PM_2.5_. Hourly mass concentrations of six heavy metals (Co, Ni, Cd, Cu, Ag, Ba) in PM_10_ and PM_2.5_ from May 1 to Jun 30, 2019 in Wuhan were monitored by the Wuhan atmospheric supersite. We conducted the health risks assessment for children and adults separately according to the guidelines of the United States Environmental Protection Agency (U.S. EPA). The exposure concentrations (EC) was evaluated to assess the exposure level of heavy metals via the inhalation route. Hazard quotient (HQ) and carcinogenic risk (CR) was evaluated respectively to estimate the non-carcinogenic and carcinogenic risks. Considering Co is Class 2B carcinogen, and Ni and Cd are Class I carcinogens (IARC (International Agency for Research on Cancer), 2011). These elements have both carcinogenic and non-carcinogenic characteristics (U.S. EPA, 2019). Therefore, both values of HQ and CR for Co, Ni and Cd were calculated in the present study. For Cu, Ag and Ba, only HQ was calculated referring to non-carcinogenic risks. The equations we used are as follows (U.S. EPA, 2011):

(1) $$\text{EC}=\frac{C\times \mathrm{ET}\times \mathrm{EF}\times \mathrm{ED}}{\mathrm{ATn}}$$

(2) HQ= EC/(RfC×1000 ug/mg)

(3) CR=IUR×EC

Where C: heavy metal concentration in PM_10_ or PM_2.5_ (ug/m^3^), ET: exposure time (hours/day), EF: exposure frequency (in the present study 350 days/year is used), ED: exposure duration (children: 6 years; adult: 24 years), ATn: averaging time (for non-carcinogens: ATn=ED×365 days/year×24 h/day; for carcinogens: ATn=70 years×365 days/year×24 h/day), RfC: inhalation reference concentration (mg/m^3^), IUR: inhalation unit risk ((ug/m3)^-1^) (U.S. EPA, 2011). The RfC, IUR were recommended by the U.S. EPA.

The hazard index (HI) was also calculated to further evaluate the accumulative non-carcinogenic risk induced by multi-metals, which referring to the summation of HQ of all heavy metals. The HQ or HI larger than 1 indicates individual’s non-carcinogenic risk, and the CR larger than 1 × 10^-4^ indicated individual’s increased risk in developing any type of cancer.

## 3 Results and discussion

### 3.1 Exposure concentrations of heavy metals in two different exposure scenarios

The 4-hour mean mass concentrations of heavy metals in PM_10_ and PM_2.5_ in two different exposure scenarios are shown in Table 1. Concentrations of Ag and Ba in PM_10_ and Co, Ag, Ba in PM_2.5_ in two exposure scenarios were below the detectable limit. However, exposure concentrations of some heavy metals, especially Ni and Cd in PM_2.5_ in the high-exposure scenario were remarkably higher than those in the low-exposure scenario (e.g. Ni: 83.08 *v.s.* 21.84 ng/m^3^, Cd: 59.45 *v.s.* 20.93 ng/m^3^). Moreover, in two exposure scenarios, levels of ambient temperature and relative humidity are very similar (29.25 ℃ and 61.00% *v.s.* 30.75 ℃ and 78.50%), which meant that they were not likely to be associated with differences in health outcomes.

**Table 1:** Averages concentrations of heavy metals and cardiovascular biomarkers at two different exposure scenarios.

Pollutants/Biomarkers, (Units)	Low-exposure scenario Mean ±SD	High-exposure scenario Mean ± SD
**PM**_**10**_ (ug/m^3^)^a^	19.00 ± 3.56	64.50 ± 7.41
Co (ng/m^3^)	0.13 ± 0.08	0.38 ± 0.04
Ni (ng/m^3^)	0.15 ± 0.05	0.20 ± 0.12
Cd (ng/m^3^)	6.61 ± 1.98	14.11 ± 3.45
Cu (ng/m^3^)	0.90 ± 0.07	3.18 ± 0.33
**PM**_**2.5**_ (ug/m^3^)^b^	11.75 ± 5.50	46.75 ± 7.76
Ni (ng/m^3^)	21.84 ± 42.96	83.08 ± 94.61
Cd (ng/m^3^)	20.93 ± 26.35	59.45 ± 46.30
Temperature (*℃)*	29.25 ± 1.26	30.75 ± 0.96
Relative humidity (%)	61.00 ± 4.55	78.50 ± 1.29
*Cardiovascular biomarkers*		
WBC, (10^^9^/L)	6.94 ± 1.50	6.90 ± 1.46
RBC, (10^^12^/L)	6.42 ± 0.54	4.60 ± 0.50
HGB, (g/L)*	326.39 ± 13.21	335.45 ± 12.43
PLT, (10^^9^/L)	285.87 ± 65.57	277.19 ± 60.19
ANC, (10^^9^/L)	4.01 ± 1.26	4.07 ± 1.11
LYM, (10^^9^/L)	2.48 ± 0.67	2.31 ± 0.60
MONO, (10^^9^/L)*	0.31 ± 0.08	0.36 ± 0.11
hs-CRP, (mg/L)	1.05 (0.99)	1.18 (2.16)
SBP, (mmHg)	107.00 ± 13.54	107.35 ± 11.93
DBP, (mmHg)	72.65 ± 9.93	72.22 ± 9.84
HR, (rate/min)*	78.61 ± 11.31	86.29 ± 13.37

a Concentrations of Ag and Ba in PM_10_ from 8:00–12:00 in two exposure scenarios were below the detectable limit.

b Concentrations of Co, Ag and Ba in PM_2.5_ from 8:00–12:00 in two exposure scenarios were below the detectable limit.

* Concentration difference of the biomarker was statistically significant.

### 3.2 Characteristics of participants

One participant only completed the low-exposure scenario was excluded. Eventually, a total of 62 serum samples collected from 31 participants (11 males and 20 females) were used for metabolomics analysis. The average age and BMI of the 31 participants were 22.6 (standard deviation (SD)= 2.5) years old and 21.8 (SD=3.4) kg/m^2^. P values from paired-*t*-test for HGB, MONO and HR were less than 0.05, and those 3 biomarkers were selected as significant cardiovascular biomarkers in the study. Higher concentrations of hs-CRP were also identified in the high-exposure scenario (1.18 *v.s.*1.05 mg/L), although no statistically significant difference was found (Table 1). Studies have reported heavy metals in PM can induce ROS and DNA damage (Zhao et al., 2019), which might explain the slightly increased of cardiovascular biomarkers in serum when exposed to higher levels of heavy metals in our study.

### 3.3 Alterations of serum metabolites

**Fig. 1:**
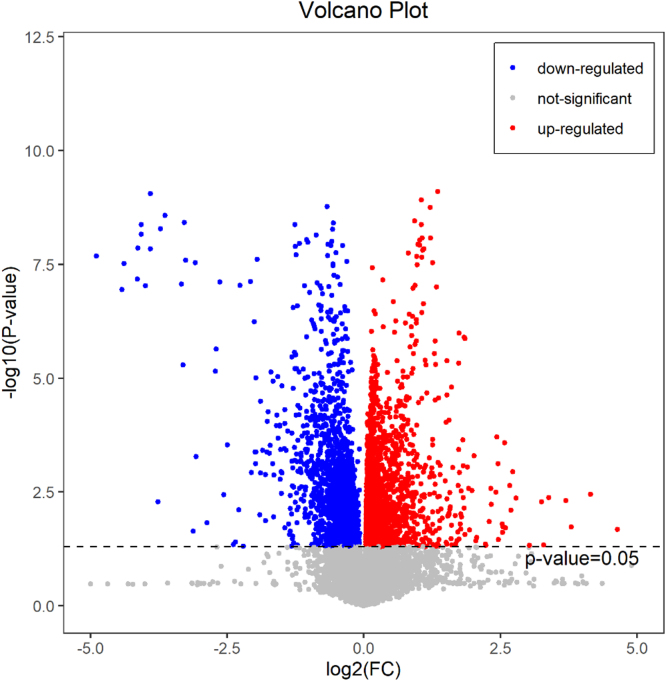
Metabolites associated with different heavy metal exposure scenarios.

In total, 4473 metabolites were identified in serum samples (Fig. 1). The scoring plot of PCA showed that the QC samples were aggregated closely, indicating our results were stable and reproducible (Figure A.1). Besides, the plot of OPLS-DA displayed a separation of serum metabolites between high-exposure scenario and low-exposure scenario (Figure A.2).

**Fig. 2:**
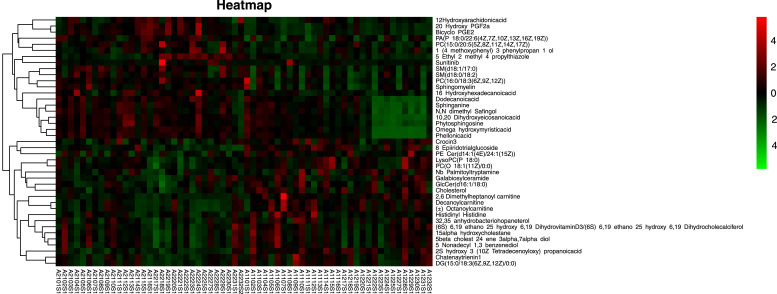
Heatmap of differential metabolites associated with significant cardiovascular biomarkers and heavy metal exposure scenarios. The color of grids from green to red indicates the expression abundance of metabolites from low to high. Grids in green indicate a low expression abundance of differential metabolites, while those in red indicate a high expression abundance.

Based on the established criteria, a total of 41 differential metabolites were identified to be related to significant cardiovascular biomarkers and short-term exposure to heavy metals, such as increased 20-Hydroxy-PGF2a, PC(15:0/20:5(5Z,8Z,11Z,14Z,17Z)), SM(d18:1/17:0), Sphingomyelin, and decreased GlcCer(d16:1/18:0) and Galabiosylceramide (Table 2, Fig. 2). Among them, 31 metabolites belong to lipids and lipid-like molecules. Other 10 metabolites belong to organic acids and derivatives, organic nitrogen compounds, organoheterocyclic compounds, phenylpropanoids and polyketides, benzenoids and unclassified molecules.

**Table 2:** Differential metabolites associated with significant cardiovascular biomarkers and heavy metal exposure scenarios.

Super class	Class	Metabolites	m/z	RT (min)	Ion model	VIP	HGB			MONO			HR			Heavy metal exposures		FC
							P	FDR		P	FDR		P	FDR		P	FDR	
Lipids and lipid-like molecules	Fatty Acyls	2,6 Dimethylheptanoyl carnitine	302.232	6.482	pos	1.249	0.000	0.000		0.019	0.252		0.260	0.558		0.000	0.000	0.562
		20-Hydroxy-PGF2a	369.228	6.917	neg	2.454	0.000	0.000		0.000	0.012		0.011	0.103		0.000	0.000	1.903
		Bicyclo-PGE2	335.221	6.986	pos	1.670	0.000	0.000		0.000	0.012		0.017	0.124		0.000	0.000	2.075
		2S-hydroxy-3-(10Z-Tetradecenoyloxy)-propanoicacid	646.452	13.577	pos	1.774	0.001	0.016		0.070	0.369		0.075	0.266		0.000	0.000	0.517
		Chatenaytrienin1	557.457	15.942	neg	1.274	0.000	0.004		0.038	0.306		0.049	0.221		0.000	0.000	0.738
		10,20-Dihydroxyeicosanoicacid	362.326	7.870	pos	1.419	0.001	0.014		0.015	0.252		0.303	0.602		0.000	0.000	1.559
		Decanoylcarnitine	316.248	7.279	pos	3.174	0.007	0.043		0.058	0.354		0.023	0.146		0.000	0.000	0.563
		Dodecanoic acid	218.211	5.630	pos	1.377	0.001	0.014		0.022	0.252		0.232	0.513		0.000	0.001	1.392
		Omega-hydroxy myristic acid	262.237	5.778	pos	1.209	0.001	0.016		0.023	0.252		0.210	0.481		0.000	0.001	1.574
		Phellonic acid	374.362	9.469	pos	1.001	0.001	0.017		0.022	0.252		0.304	0.602		0.000	0.001	1.619
		DG(15:0/18:3(6Z,9Z,12Z)/0:0)	557.457	14.843	neg	2.026	0.001	0.014		0.114	0.452		0.064	0.248		0.001	0.002	0.773
		12 Hydroxy arachidonic acid	319.228	10.532	neg	1.731	0.008	0.045		0.020	0.252		0.539	0.792		0.006	0.008	1.383
		16-Hydroxy hexadecenoic acid	290.269	7.828	pos	1.258	0.007	0.044		0.570	0.871		0.085	0.275		0.008	0.010	1.343
	Glycerophospholipids	PC(15:0/20:5(5Z,8Z,11Z,14Z,17Z))	748.526	17.451	pos	1.395	0.000	0.004		0.038	0.306		0.059	0.234		0.000	0.000	1.956
		LysoPC(P-18:0)	552.367	10.714	neg	1.658	0.001	0.017		0.027	0.258		0.001	0.077		0.000	0.001	0.850
		PC(O-18:1(11Z)/0:0)	508.376	10.722	pos	3.510	0.003	0.030		0.088	0.404		0.003	0.082		0.001	0.001	0.849
		PA(P-18:0/22:6(4Z,7Z,10Z,13Z,16Z,19Z))	750.543	12.362	pos	2.941	0.003	0.030		0.009	0.252		0.305	0.602		0.001	0.002	1.408
		PC(16:0/18:3(6Z,9Z,12Z))	756.553	12.362	pos	19.622	0.005	0.040		0.037	0.306		0.006	0.093		0.002	0.002	1.474
	Prenollipids	Crocin 3	697.268	5.328	neg	1.794	0.006	0.041		0.133	0.491		0.667	0.860		0.000	0.000	0.807
		8-Epiiridotrial glucoside	343.140	5.378	neg	1.482	0.001	0.014		0.316	0.754		0.237	0.514		0.000	0.000	0.609
		32,35-anhydrobacteriohopaneterol	573.452	12.850	neg	1.104	0.001	0.017		0.138	0.498		0.032	0.183		0.001	0.001	0.818
	Sphingolipids	GlcCer(d16:1/18:0)	700.572	15.218	pos	1.006	0.004	0.034		0.299	0.750		0.000	0.023		0.000	0.001	0.788
		SM(d18:1/17:0)	717.589	12.775	pos	6.320	0.004	0.034		0.111	0.452		0.035	0.191		0.001	0.001	1.606
		Galabiosylceramide	862.624	14.726	pos	1.812	0.003	0.031		0.120	0.458		0.004	0.093		0.002	0.003	0.828
		Sphingomyelin	729.590	12.362	pos	14.948	0.006	0.041		0.037	0.306		0.006	0.094		0.003	0.004	1.484
		SM(d18:0/18:2)	773.582	11.501	neg	5.765	0.007	0.043		0.058	0.354		0.005	0.093		0.004	0.006	1.492
		PE-Cer(d14:1(4E)/24:1(15Z))	715.574	12.362	pos	1.578	0.007	0.043		0.680	0.880		0.978	0.995		0.005	0.007	0.713
	Steroids and steroid derivatives	Cholesterol	369.351	17.266	pos	4.400	0.008	0.046		0.211	0.617		0.000	0.023		0.001	0.001	0.824
	Sterol lipids	15alpha-hydroxycholestane	449.364	12.962	neg	1.529	0.003	0.030		0.210	0.617		0.203	0.480		0.010	0.012	0.874
		5beta-cholest-24-ene-3alpha,7alpha-diol	447.348	12.806	neg	2.040	0.002	0.025		0.368	0.803		0.325	0.628		0.015	0.017	0.900
		(6 S)-6,19-ethano-25-hydroxy-6,19-DihydrovitaminD3/(6 S)-6,19-ethano-25-hydroxy-6,19-Dihydrocholecalciferol	473.363	12.976	neg	1.567	0.003	0.031		0.258	0.675		0.535	0.792		0.017	0.018	0.863
Organic acids and derivatives	Carboxylic acids and derivatives	Histidinyl-Histidine	585.270	7.028	pos	4.425	0.003	0.030		0.364	0.803		0.790	0.894		0.004	0.005	0.737
Organic nitrogen compounds	Organonitrogen compounds	Phytosphingosine	318.300	7.814	pos	2.400	0.001	0.016		0.043	0.318		0.696	0.878		0.000	0.001	1.430
		Sphinganine	302.305	8.617	pos	1.082	0.002	0.026		0.050	0.348		0.470	0.757		0.001	0.001	1.395
Organoheterocyclic compounds	Azoles	5-Ethyl-2-methyl-4-propylthiazole	187.126	7.279	pos	2.397	0.000	0.004		0.003	0.193		0.070	0.259		0.000	0.001	1.338
	Indoles and derivatives	Nb-Palmitoyltryptamine	416.364	16.180	pos	1.301	0.023	0.077		0.010	0.252		0.002	0.077		0.009	0.011	0.834
		Sunitinib	397.205	8.165	neg	1.163	0.004	0.034		0.063	0.363		0.536	0.792		0.013	0.015	1.743
Phenylpropanoids and polyketides	Linear1,3-diarylpropanoids	1-(4-methoxyphenyl)-3-phenylpropan-1-ol	502.293	8.532	pos	1.079	0.002	0.027		0.216	0.617		0.992	0.997		0.012	0.014	1.292
Benzenoids	Phenols	5-Nonadecyl-1,3-benzenediol	421.332	12.371	neg	1.272	0.007	0.044		0.403	0.814		0.337	0.633		0.036	0.038	0.918
Unclassified	Unclassified	(±)-Octanoylcarnitine	288.217	6.236	pos	2.374	0.009	0.049		0.044	0.318		0.025	0.146		0.000	0.000	0.590
		N,N-dimethyl-Safingol	330.336	9.440	pos	1.107	0.004	0.033		0.115	0.452		0.705	0.879		0.001	0.001	1.313

Our study is one of the few studies to report short-term exposure to heavy metals of PM could be associated with disturbance of cardiovascular-related metabolites in adults. Our findings support previous studies showing that higher exposure to PM leads to metabolic disturbance (Li et al., 2017b, Wang et al., 2017). For example, Li and colleagues found significant changes in lipids and fatty acids among adults exposed to higher PM_2.5_ (Li et al., 2017b). And in animal studies, Wang et al. found adult male rats treated with PM_2.5_ were correlated with changes of lipid and nucleotide metabolism (Wang et al., 2017). Similarly results also reported in another study that ApoE^−/−^ mice exposure to PM_2.5_ for 2-month could cause mice serum lipid dysregulation (Zhang et al., 2020). Disruption of lipids metabolites may lead to cardiovascular disease including obesity, dyslipidemia and atherosclerosis (van der Veen et al., 2017), showing increased risks of chronic diseases.

The sphingolipid is an important class of lipids metabolites, and have been confirmed as critical regulators of cardiovascular disease and cancer (Lemaitre et al., 2018). Compared with the low-exposure scenario, we found levels of SM(d18:1/17:0), Sphingomyelin and SM(d18:0/18:2) in serum were increased in the high-exposure scenario, while levels of GlcCer(d16:1/18:0), Galabiosylceramide and PE-Cer(d14:1(4E)/24:1(15Z)) were decreased (Table 2). Similar results were reported in animal studies. For example, Zhang et al. found the levels of sphingolipid metabolites in broncho-alveolar lavage fluid of mice were increased after PM_2.5_ instillation (Zhang et al., 2017). Besides, Zhao et al. found metabolites of serum sphingolipids differed in the PM_2.5_-exposure group from that in the control group (Zhao et al., 2019). It has been reported that circular levels of sphingolipids are independently associated with adverse cardiovascular events (Meeusen et al., 2018). The sphingolipids may be key regulators in the heavy metal exposure and cardiovascular-related early health effects.

### 3.4 Metabolic pathways analysis

**Fig. 3:**
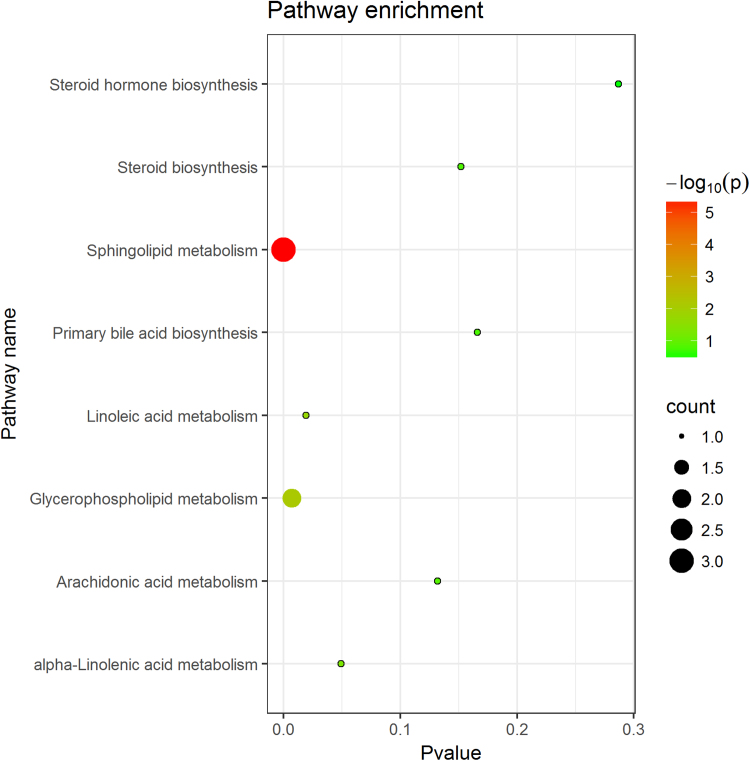
Enriched metabolic pathways for heavy metal exposures to influence cardiovascular-related early health effects.

**Fig. 4:**
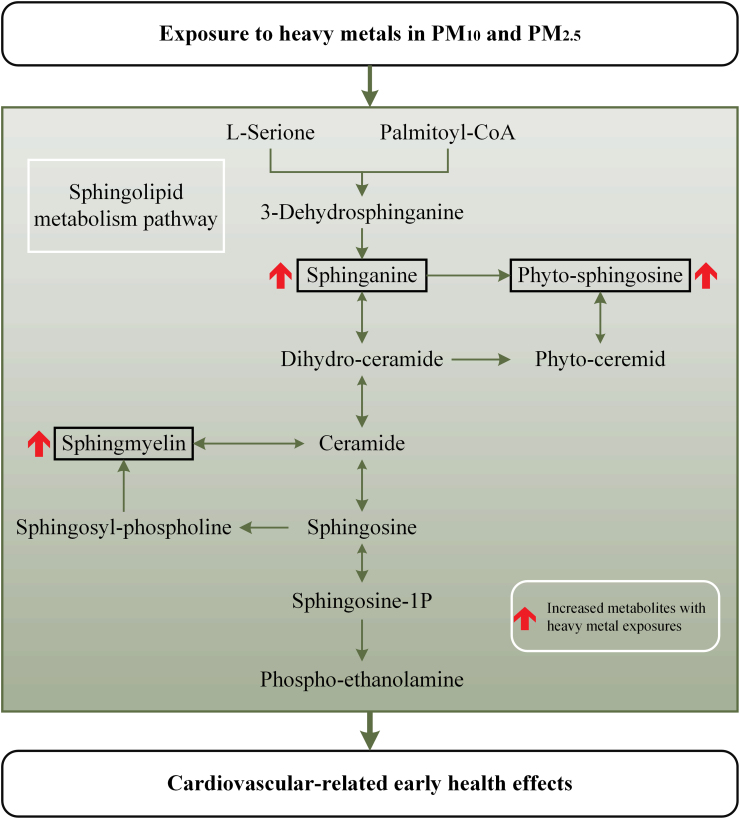
Potential mechanisms underlying cardiovascular-related early health effects of heavy metal exposures. Legends: The red arrow with solid text boxes indicates increased metabolites with heavy metal exposures. Metabolites with no text boxes were in-significantly changed metabolites, which involved in the sphingolipid metabolism pathway.

Results of metabolic pathway analysis showed the sphingolipid metabolism pathway was the main biological pathway associated with cardiovascular-related early health effects affected by heavy metal exposures (p value less than 0.05, FDR less than 0.05) (Table A.2). A total of 3 differential metabolites were integrated into the sphingolipid metabolism pathway, including Sphingomyelin, Phytosphingosine and Sphinganine (Fig. 3, Fig. 4). Evidence has shown the importance of sphingolipid metabolism in regulating cell signaling activity, apoptosis and inflammatory, and its integral structural constituents of lipid membranes (Merrill, 2011). Enhanced sphingolipid metabolism pathway has been considered as an important independent risk factor of cardiovascular disease (Yu et al., 2019, Le Barz et al., 2020, Jensen et al., 2020) and cancer (Sui et al., 2019). In mouse models, activation of sphingolipid metabolism pathway caused by an elevation in sphingomyelin contributed to the inflammatory state of the liver and increased expression of CRP (Lightle et al., 2000). Decreased plasma sphingomyelin levels was found to be associated with a lower secretion of pro-inflammatory cytokine in mouse (Li et al., 2019). Further studies on the effect of sphingolipid are needed in order to better understand the molecular mechanism between airborne heavy metal exposures and cardiovascular-related early health effects.

### 3.5 Health risks assessment

The mean mass concentrations of Co, Ni, Cd, Cu, Ag and Ba in PM_10_ and PM_2.5_ in Wuhan from May 1 to Jun 30, 2019 are listed in Table A.3. Ag has the highest mass concentrations in PM_10_ (34.47 ng/m^3^), followed by Cd (11.53 ng/m^3^), Ba (4.19 ng/m^3^), Cu (2.23 ng/m^3^), Ni (0.49 ng/m^3^) and Co (0.22 ng/m^3^). PM_2.5_ showed stronger adsorption capacity of heavy metals than PM_10_, with the concentration of Ba in PM_2.5_ as high as 422.62 ng/m^3^, followed by Cu (291.94 ng/m^3^), Cd (291.85 ng/m^3^), Ni (128.47 ng/m^3^), Ag (98.55 ng/m^3^), and Co (0.86 ng/m^3^).

The estimated non-carcinogenic risks of heavy metals are summarized in Table 3. HQ for Cd in PM_10_ and PM_2.5_ and HQ for Ni in PM_2.5_ via the inhalation route for children and adults were greater than 1, demonstrating potential non-carcinogenic risks posed by these heavy metals. And the non-carcinogenic risks of Ni and Cd may increase if potential synergy is considered between heavy metals and other toxic components (e.g., sulfate, nitrates and ammonia) in PM. It is important to notice that non-carcinogenic risks of Ni and Cd in mega cities of China were reported at acceptable levels. For example, in Nanjing, HQ for Ni in PM_2.5_ via the inhalation route was 1.09E-01, and for Cd, it was 1.87E-01 (Hu et al., 2012). In Tianjin, HQ for Ni and Cd in PM_2.5_ were also less than 1 (Zhang et al., 2015). The HI for accumulative non-carcinogenic risk of Co, Ni, Cd, Cu, Ag and Ba via the inhalation route was slightly greater than 1, indicating an accumulative non-carcinogenic risk via the inhalation route. Moreover, the HI of heavy metals via the inhalation route in PM_2.5_ were higher than that in PM_10_ (3.14E+01 *v.s.*1.17E+00), suggesting greater risks of PM_2.5_ than PM_10_.

**Table 3:** Non-carcinogenic risks from heavy metals (Co, Ni, Cu, Ag, Cd, Ba) in PM_10_ and PM_2.5_.

Species	RfC (mg/m^3^)	EC		HQ	
		Child	Adults	Child	Adults
*PM_10_*					
Co	6.00E−06	2.11E−04	2.11E−04	3.51E−02	3.51E−02
Ni	5.00E−05	4.71E−04	4.71E−04	9.42E−03	9.42E−03
Cd	1.00E−05	1.11E−02	1.11E−02	1.11E+00	1.11E+00
Cu	4.00E−02	2.14E−03	2.14E−03	5.34E−05	5.34E−05
Ag	5.00E−03	3.31E−02	3.31E−02	6.61E−03	6.61E−03
Ba	5.00E−04	4.02E−03	4.02E−03	8.03E−03	8.03E−03
**HI**				1.17E+00	1.17E+00
*PM_2.5_*					
Co	6.00E−06	8.24E−04	8.24E−04	1.37E−01	1.37E−01
Ni	5.00E−05	1.23E−01	1.23E−01	2.46E+00	2.46E+00
Cd	1.00E−05	2.80E−01	2.80E−01	2.80E+01	2.80E+01
Cu	4.00E−02	2.80E−01	2.80E−01	7.00E−03	7.00E−03
Ag	5.00E−03	9.45E−02	9.45E−02	1.89E−02	1.89E−02
Ba	5.00E−04	4.05E−01	4.05E−01	8.11E−01	8.11E−01
**HI**				3.14E+01	3.14E+01

The estimated carcinogenic risks of Co, Ni and Cd in PM_10_ and PM_2.5_ were shown in Table 4. The carcinogenic risks induced by Co, Ni and Cd in PM_10_ via the inhalation route for children and adults were at an acceptable level (CR<1 × 10^-4^). However, CR was slightly higher than 1 × 10^-4^ for Cd in PM_2.5_ via the inhalation route for adults (CR=1.73E-04), indicating that Cd in PM_2.5_ had potential risks of developing cancers among adults.

**Table 4:** Carcinogenic risks from heavy metals (Co, Ni, Cd) in PM_10_ and PM_2.5_.

Species	IUR (ug/m^3^)^-1^	EC		CR	
		Child	Adults	Child	Adults
*PM_10_*					
Co	9.00E−03	1.81E−05	7.23E−05	1.63E−07	6.51E−07
Ni	2.40E−04	4.04E−05	1.62E−04	9.69E−09	3.88E−08
Cd	1.80E−03	9.48E−04	3.79E−03	1.71E−06	6.47E−09
*PM_2.5_*					
Co	9.00E−03	7.06E−05	2.82E−04	6.36E−07	2.54E−06
Ni	2.40E−04	1.06E−02	4.22E−02	2.53E−06	1.01E−05
Cd	1.80E−03	2.40E−02	9.60E−02	4.32E−05	1.73E−04

Several limitations should be noted in our study. Firstly, the concentrations of heavy metals were estimated using data from the atmospheric supersite, which represented the region or city-level rather than the individual-level exposures. Variations in exposure to pollutants were not considered between individuals. Secondly, the disturbance of cardiovascular-related metabolites we observed in the present study was associated with heavy metal mixture. Metabolites changes affected by each single heavy metal were not identified. Thirdly, due to difficulties in measuring other components or exposures (e.g., cyclic or branched alkanes, polycyclic aromatic hydrocarbons (PAHs), carbon particles, endotoxin and inorganic substances) at the same time, we were unable to consider the potential toxic effects of them in this study. Fourthly, baseline blood samples for each participant were not collected in the present study. We were also unable to evaluate the intraday variance of biomarkers and metabolome under normal physiological state and the potential lag effects of metabolite changes, as blood samples of participants were only collected once after exposures. Finally, the standard default values, such as EF, ED, RfC and RfD, are recommended by the U.S.EPA. It has not been confirmed whether these values were proper to be used in Wuhan population.

## 4 Conclusions

In conclusion, this research suggested that serum cardiovascular-related metabolites disturbance existed in healthy adults exposed to heavy metals, and those diﬀerential metabolites were associated with activation of sphingolipid metabolism pathway. The accumulative non-carcinogenic risk and potential carcinogenic risk of heavy metals through the inhalation route was beyond the acceptable level for individuals in Wuhan. Ni and Cd most influenced to potential health risks. The environmental authority is advised to pay more attention to heavy metal components of particulate matter.

## CRediT authorship contribution statement

**Feifei Liu**: Investigation, Methodology, Validation, Formal analysis, Data curation, Writing - original draft. **Xiaolu Chen**: Investigation, Methodology, Validation, Formal analysis, Data curation, Writing - original draft. **Yisi Liu**: Conceptualization, Software, Writing - review & editing. **Zhiping Niu**: Investigation, Methodology, Software. **Hong Tang**: Investigation, Methodology, Software. **Shuyuan Mao**: Investigation, Formal analysis. **Na Li**: Investigation, Formal analysis. **Gongbo Chen**: Conceptualization, Supervision, Project administration, Writing - review & editing. **Hao Xiang**: Investigation, Supervision, Validation, Project administration, Resources.

## Declaration of Competing Interest

The authors declare that they have no known competing financial interests or personal relationships that could have appeared to influence the work reported in this paper.
