# Supplementary material for: Serum cardiovascular-related metabolites disturbance exposed to different heavy metal exposure scenarios
Source: J Hazard Mater. 2021 Aug 5;415:125590. doi: 10.1016/j.jhazmat.2021.125590 (PMC8204224; doi:10.1016/j.jhazmat.2021.125590)
Supplement: Supplementary file 1 — Supplementary material [file mmc1.docx]

**Appendices**

Serum cardiovascular-related metabolites disturbance exposed to different heavy metal exposure scenarios

Feifei Liu^†,‡,1^, Xiaolu Chen^†,‡,1^, Yisi Liu^&^, Zhiping Niu^†,‡^, Hong Tang^†,‡^, Shuyuan Mao^†,‡^, Na Li^†,‡^, Gongbo Chen^†,‡,*^, Hao Xiang^†,‡,*^

^†^ Department of Global Health, School of Health Sciences, Wuhan University, 115# Donghu Road, Wuhan, 430071, China.

^‡^ Global Health Institute, School of Health Sciences, Wuhan University, 115# Donghu Road, Wuhan, 430071, China.

^&^ Department of Environmental and Occupational Health Sciences, University of Washington, Seattle, WA 98105, USA.

^1^ These authors contributed equally to this work.

* Corresponding author:

Dr. Gongbo Chen, E-Mail: chen.gongbo@whu.edu.cn.

Dr. Hao Xiang, E-Mail: xianghao@whu.edu.cn.

Table A.1 STROBE checklist of items that should be included in reports of observational studies

| **Item** | No | Recommendation | Page |
| --- | --- | --- | --- |
| **Title and abstract** | 1 | (*a*) Indicate the study’s design with a commonly used term in the title or the abstract | 1-2 |
|  |  | (*b*) Provide in the abstract an informative and balanced summary of what was done and what was found | 2 |
| Introduction | | |  |
| Background/rationale | 2 | Explain the scientific background and rationale for the investigation being reported | 3-4 |
| Objectives | 3 | State specific objectives, including any prespecified hypotheses | 5 |
| Methods | | |  |
| Study design | 4 | Present key elements of study design early in the paper | 5-6 |
| Setting | 5 | Describe the setting, locations, and relevant dates, including periods of recruitment, exposure, follow-up, and data collection | 5-8 |
| Participants | 6 | (*a*) *Cohort study*—Give the eligibility criteria, and the sources and methods of selection of participants. Describe methods of follow-up  *Case-control study*—Give the eligibility criteria, and the sources and methods of case ascertainment and control selection. Give the rationale for the choice of cases and controls  *Cross-sectional study*—Give the eligibility criteria, and the sources and methods of selection of participants | 5-8 |
|  |  | (*b*) *Cohort study*—For matched studies, give matching criteria and number of exposed and unexposed  *Case-control study*—For matched studies, give matching criteria and the number of controls per case | NA |
| Variables | 7 | Clearly define all outcomes, exposures, predictors, potential confounders, and effect modifiers. Give diagnostic criteria, if applicable | 8-12 |
| Data sources/ measurement | 8* | For each variable of interest, give sources of data and details of methods of assessment (measurement). Describe comparability of assessment methods if there is more than one group | 8-12 |
| Bias | 9 | Describe any efforts to address potential sources of bias | NA |
| Study size | 10 | Explain how the study size was arrived at | NA |
| Quantitative variables | 11 | Explain how quantitative variables were handled in the analyses. If applicable, describe which groupings were chosen and why | 8-12 |
| Statistical methods | 12 | (*a*) Describe all statistical methods, including those used to control for confounding | 8-12 |
|  |  | (*b*) Describe any methods used to examine subgroups and interactions | NA |
|  |  | (*c*) Explain how missing data were addressed | NA |
|  |  | (*d*) *Cohort study*—If applicable, explain how loss to follow-up was addressed  *Case-control study*—If applicable, explain how matching of cases and controls was addressed  *Cross-sectional study*—If applicable, describe analytical methods taking account of sampling strategy | 8-12 |
|  |  | (*e*) Describe any sensitivity analyses | NA |

Continued on next page

| Results | | |  |
| --- | --- | --- | --- |
| Participants | 13* | (a) Report numbers of individuals at each stage of study—eg numbers potentially eligible, examined for eligibility, confirmed eligible, included in the study, completing follow-up, and analysed | 12-13 |
|  |  | (b) Give reasons for non-participation at each stage | 12 |
|  |  | (c) Consider use of a flow diagram | NA |
| Descriptive data | 14* | (a) Give characteristics of study participants (eg demographic, clinical, social) and information on exposures and potential confounders | 12-13 |
|  |  | (b) Indicate number of participants with missing data for each variable of interest | NA |
|  |  | (c) *Cohort study*—Summarise follow-up time (eg, average and total amount) | NA |
| Outcome data | 15* | *Cohort study*—Report numbers of outcome events or summary measures over time | 11-16 |
|  |  | *Case-control study—*Report numbers in each exposure category, or summary measures of exposure | *NA* |
|  |  | *Cross-sectional study—*Report numbers of outcome events or summary measures | *NA* |
| Main results | 16 | (*a*) Give unadjusted estimates and, if applicable, confounder-adjusted estimates and their precision (eg, 95% confidence interval). Make clear which confounders were adjusted for and why they were included | 12-17 |
|  |  | (*b*) Report category boundaries when continuous variables were categorized | NA |
|  |  | (*c*) If relevant, consider translating estimates of relative risk into absolute risk for a meaningful time period | NA |
| Other analyses | 17 | Report other analyses done—eg analyses of subgroups and interactions, and sensitivity analyses | 12-17 |
| Discussion | | |  |
| Key results | 18 | Summarise key results with reference to study objectives | 17-18 |
| Limitations | 19 | Discuss limitations of the study, taking into account sources of potential bias or imprecision. Discuss both direction and magnitude of any potential bias | 17 |
| Interpretation | 20 | Give a cautious overall interpretation of results considering objectives, limitations, multiplicity of analyses, results from similar studies, and other relevant evidence | 12-17 |
| Generalisability | 21 | Discuss the generalisability (external validity) of the study results | NA |
| Other information | | |  |
| Funding | 22 | Give the source of funding and the role of the funders for the present study and, if applicable, for the original study on which the present article is based | 18 |

*Give information separately for cases and controls in case-control studies and, if applicable, for exposed and unexposed groups in cohort and cross-sectional studies.

Table A.2 Metabolic pathways associated with cardiovascular-related early health effects of heavy metal exposures

| Pathway Name | Match Status | p-value | FDR |
| --- | --- | --- | --- |
| Sphingolipid metabolism | 3/21 | 4.182E-6 | 0.035 |
| Glycerophospholipid metabolism | 2/36 | 0.007 | 0.312 |
| Linoleic acid metabolism | 1/5 | 0.019 | 0.538 |
| alpha-Linolenic acid metabolism | 1/13 | 0.049 | 1.0 |
| Arachidonic acid metabolism | 1/36 | 0.132 | 1.0 |
| Steroid biosynthesis | 1/42 | 0.152 | 1.0 |
| Primary bile acid biosynthesis | 1/46 | 0.166 | 1.0 |
| Steroid hormone biosynthesis | 1/85 | 0.287 | 1.0 |

Table A.3 The descriptive statistics of heavy metal components in PM_10_ and PM_2.5_ (ng/m^3^) of Wuhan, China

| Species | PM_10_ |  | PM_2.5_ |
| --- | --- | --- | --- |
|  | Mean ± SD |  | Mean ± SD |
| Co | 0.22 ± 0.13 |  | 0.86 ± 2.58 |
| Ni | 0.49 ± 0.47 |  | 128.47 ± 202.43 |
| Cd | 11.53 ± 6.79 |  | 291.85 ± 394.03 |
| Cu | 2.23 ± 1.26 |  | 291.94 ± 423.16 |
| Ag | 34.47 ± 37.37 |  | 98.55 ± 137.63 |
| Ba | 4.19 ± 2.81 |  | 422.62 ± 3067.27 |


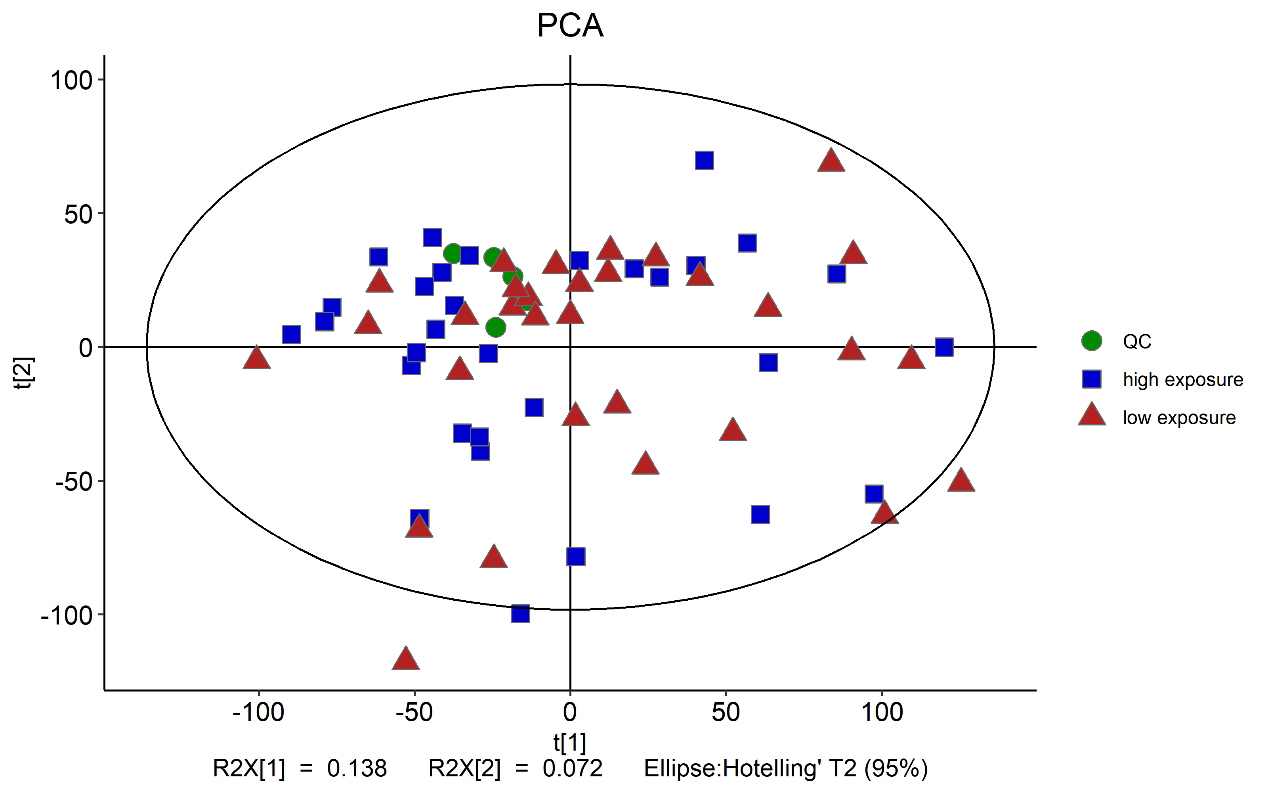


Figure A.1 Scoring plot of principal component analysis


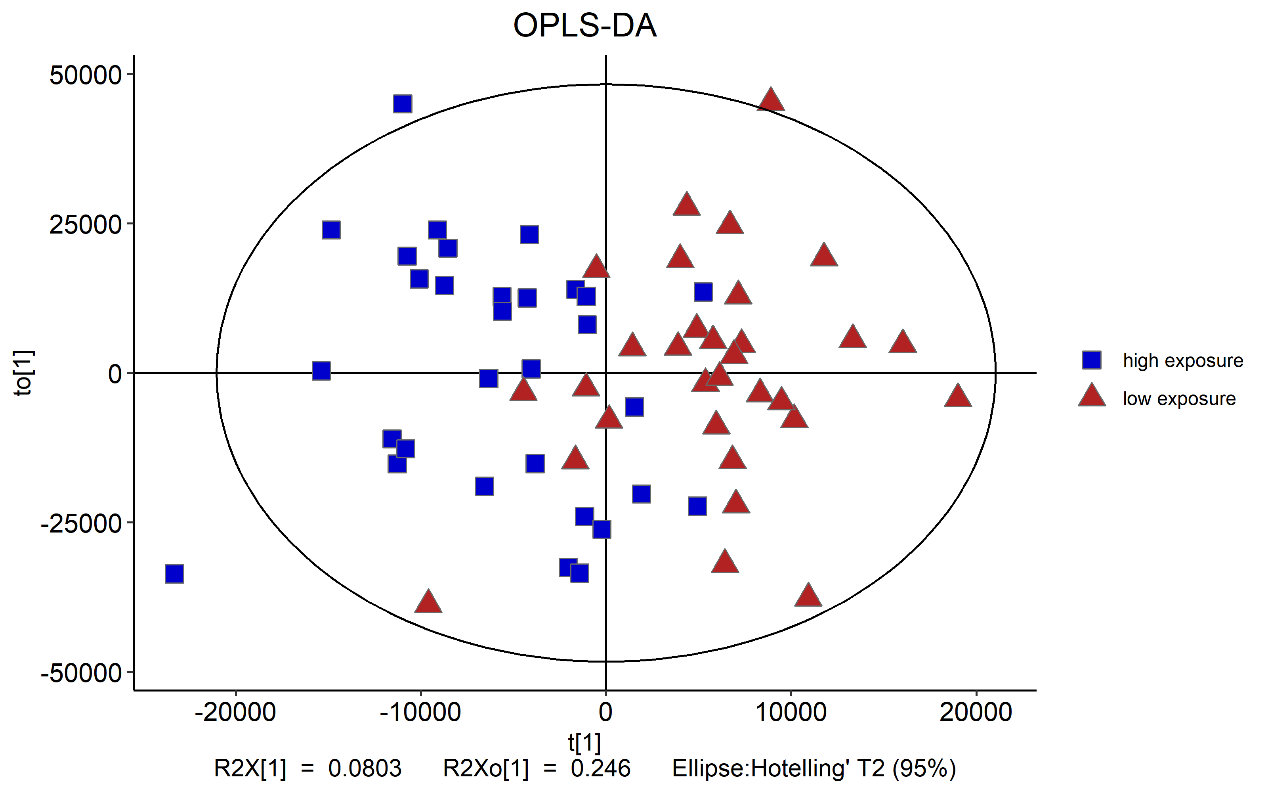


Figure A.2 Scoring plot of orthogonal partialleast-squares-discriminant analysis
